# Hearing Abilities in Children with Perinatally Acquired HIV, Children Perinatally Exposed to HIV but Uninfected, and Children Unexposed to HIV

**DOI:** 10.3390/audiolres15060170

**Published:** 2025-12-05

**Authors:** Peter Torre, Haley Elliott, Zhongli J. Zhang, Tzy-Jyun Yao, Barbara Laughton

**Affiliations:** 1 School of Speech, Language, and Hearing Sciences, San Diego State University, San Diego, CA 92182, USA; 2 Tygerberg Hospital, Cape Town 7594, South Africa; haley.elliott13@gmail.com; 3 Center for Biostatistics in AIDS Research, Harvard University, Boston, MA 02115, USA; zzhang@sdac.harvard.edu (Z.J.Z.); tyao@sdac.harvard.edu (T.-J.Y.); 4 Department of Paediatrics and Child Health, Stellenbosch University, Cape Town 7602, South Africa; bl2@sun.ac.za

**Keywords:** children, hearing, HIV, perinatal

## Abstract

**Background/Objectives**: Children with perinatal HIV (PHIV) are more at risk for hearing loss than HIV-unexposed (HU) children. Due to medical advances maternal HIV transmission to newborns is decreasing, but in children with perinatal HIV exposure, uninfected (PHEU) is increasing. The objectives were to evaluate (1) pure-tone audiometry and cochlear and auditory neural function in children with perinatally acquired HIV (PHIV), children with perinatal HIV exposure but uninfected (PHEU), and HIV-unexposed (HU) children and (2) differences in hearing measures for children with PHIV according to HIV disease severity. **Methods**: Three hundred and thirty-three children (105 PHIV [58 girls, 47 boys], 101 PHEU [51 girls, 50 boys], and 127 HU [65 girls, 62 boys]), aged 11–14 years, completed a hearing assessment that included a hearing-related questionnaire, otoscopy, tympanometry, pure-tone thresholds, distortion product otoacoustic emissions (DPOAEs) for cochlear function, and auditory brainstem responses (ABRs) for neural function. **Results**: Pure-tone thresholds, DPOAE, and ABR measures were similar in the three groups. Children with PHIV had a higher prevalence of hearing loss compared to children with PHEU and HU children. Children with PHIV and greater historical HIV disease severity had similar hearing, DPOAEs, and ABRs to those with lesser HIV disease severity. **Conclusions**: In utero HIV acquisition or HIV exposure might not affect the cochlear and neural function up to the level of the brainstem. Children with PHIV had a higher prevalence of hearing loss; it is possible there is a difference in central auditory processing across the three groups of children. Hearing loss identification is important since it may impact social and educational development.

## 1. Introduction

The literature on the association between human immunodeficiency virus (HIV) and hearing is growing, particularly in children [1,2,3,4,5]. Children with perinatally acquired HIV (PHIV) are at higher risk for hearing loss compared to children without HIV [1,2,3,4]. Because of advances in medications preventing maternal HIV transmission to neonates, the number of newborns with PHIV has been substantially reduced [6,7]. The result is an increasing number of children born with perinatal HIV exposure but uninfected (PHEU). Children with PHEU were exposed in utero to HIV, maternal antiretroviral therapy (ART), and the maternal inflammatory markers, some of which can cross the placenta [8] and may impact fetal development. It is unclear how in utero HIV and ART exposure impacts the auditory system for children with PHEU.

Hearing tests, like pure-tone threshold audiometry, require an individual to respond to an auditory stimulus. These measures evaluate the entire auditory system from the cochlea to the auditory cortex. Physiologic measures of auditory function do not require responses from individuals and are used to evaluate specific structures within the auditory system for subclinical damage. Otoacoustic emissions (OAEs) measure outer hair cell integrity within the cochlea to an external stimulus [9]. Auditory brainstem responses (ABRs) are used to evaluate the afferent neural integrity of auditory nerve fibers from the cochlea to the level of the brainstem via electrodes placed on the head [10].

Little OAE data exists regarding children and adolescents with PHIV or PHEU. No significant association was reported between HIV status and distortion product OAEs (DPOAEs) in adolescents with PHIV and PHEU [11], but among adolescents with PHIV, higher viral load (≥400 copies/mL) was associated with poorer DPOAEs [11]. Conversely, children with HIV had significantly poorer DPOAEs at most frequencies tested compared to children unexposed to HIV (HU) [12]. For ABR data, children with HIV have poor ABR peak morphology and low peak amplitude [13], suggesting a lack of neural synchrony. And children with an AIDS diagnosis were found to have impaired auditory neural function despite normal hearing [14]. Children with HIV may have HIV-related central nervous system disorders [15], which may include the auditory nervous system. As a result, obtaining ABR measures is warranted when evaluating the auditory system.

To summarize, previous research has shown lower hearing sensitivity in children with HIV, but there are few studies on the effects of HIV on cochlear and auditory neural function and very little research on hearing in children with PHEU. To investigate the effects of HIV on the auditory system, the specific objectives of this study were to (1) compare pure-tone audiometry, DPOAE, and ABR measures in children with PHIV, PHEU, and HU children and (2) evaluate differences in pure-tone audiometry, DPOAE, and ABR measures for children with PHIV but with different HIV disease severity.

## 2. Materials and Methods

### 2.1. Participants

Children between 11 and 14 years old were prospectively enrolled. Most children were recruited from studies within the Family Centre for Research with Ubuntu (FAMCRU), a clinical research center that focuses on clinical studies of HIV and tuberculosis. The primary site is located at Tygerberg Academic Hospital of Stellenbosch University in Cape Town, South Africa. Additional children without HIV were recruited from neighborhoods with similar demographics. For children who were not enrolled in an existing FAMCRU study, the mother received an HIV test to confirm her HIV status. To confirm that children with PHEU and HU children were negative, they were tested using two rapid HIV antibody tests from different manufacturers.

### 2.2. Procedures

#### 2.2.1. Informed Consent

All procedures were approved by the San Diego State University, Harvard T. H. Chan School of Public Health Institutional Review Boards, and the Health Research Ethics Committee at Stellenbosch University (Reg No: M19/04/016). The protocol, risks, and benefits of the study were explained in person to children and caregivers in their preferred language by a research assistant. Written informed consent was obtained from the child’s parent or legal guardian and participants provided assent.

#### 2.2.2. Questionnaires

Research assistants administered a questionnaire regarding the child’s hearing health to the parent/caregiver using their preferred language. Questions included the following: history of middle ear infections; trauma to the head or ear(s); caregiver perception of any hearing problems, noise exposure, previous hearing aid use; and other medical history. Child demographic questions included date of birth, sex, race, primary language used, educational level, and academic performance at school. Caregiver’s characteristics questions included educational level, current occupational status, and annual household income.

#### 2.2.3. Otoscopy and Tympanometry

The audiologist was blinded to PHIV exposure of children throughout hearing testing. In a clinic room, the audiologist performed bilateral otoscopy on each ear of the child. Presence of cerumen in the external auditory canal, color and position of the tympanic membrane, and presence of perforations were documented. After otoscopy, the audiologist obtained bilateral tympanograms to ensure proper transfer of acoustic stimuli through the middle ear. If the audiologist detected active otitis media based on otoscopy and tympanometry, testing was halted, and the child was referred to the Ear, Nose, and Throat clinic within Tygerberg Hospital. The child was rescheduled for testing once the infection resolved.

#### 2.2.4. Pure-Tone Audiometry

Audiometric testing was performed in sound-treated rooms within the audiology clinic. Pure-tone audiometry using supra-aural headphones (Telephonics TDH−50 P) was completed using procedures recommended by the American Speech-Language-Hearing Association [16]. Air-conduction (AC) thresholds were obtained at octave frequencies from 250–8000 Hz in both ears. If AC thresholds were ≥20 decibels of hearing level (dB HL), bone-conduction (BC) was completed, and BC thresholds were determined for 500–4000 Hz.

#### 2.2.5. Distortion Product Otoacoustic Emissions and Auditory Brainstem Responses

DPOAEs were measured with a probe assembly placed in the ear canal and using two primary frequencies (*f*_1_ and *f*_2_, where *f*_2_ > *f*_1_) at *f*_2_/*f*_1_ = 1.22, *f*_2_ swept from 1078.1 through 7230.5 Hz, with primary levels (L_1_, L_2_) of 65/55 dB SPL. The child was instructed to remain as quiet as possible and that they did not need to respond. ABRs were obtained using alternating rarefaction and condensation clicks through insert earphones at a rate of 11.1/sec and at 75 dB nHL. Electrodes were placed to the child’s high forehead (Fz), the right and left earlobes, and the ground at the lower forehead (Fpz). Each electrode site was prepped with an alcohol wipe, skin exfoliant, then conductive paste and finally secured with medical tape. The child reclined on a cot and was told that they could fall asleep. Recording parameters for each ear were a minimum of two sweeps of 2000 rarefaction clicks and then two sweeps of 2000 condensation clicks. At least two ABRs were completed to ensure waveform reliability. Only the rarefaction ABR data were used for analyses and to compare to norms.

#### 2.2.6. Neurocognitive Abilities

The Kaufmann Assessment Battery for Children Second Edition (KABC-II) was administered to participants by trained research assistants, under supervision of an Educational Psychologist, in the participants’ preferred language [17].

#### 2.2.7. Statistical Analyses

Summary statistics for demographics and hearing outcomes were calculated by PHIV exposure and compared by Kruskal–Wallis test for continuous variables and Fisher’s exact test or Chi-Square test as appropriate for categorical variables. Hearing loss was defined as a worse ear pure-tone average (PTA) of 500, 1000, 2000, and 4000 Hz > 15 dB. Prevalence of hearing loss was estimated for each group with exact 95% confidence intervals. Ordinary least square linear models were used to evaluate the association between PHIV exposure and worse ear PTA (as a continuous variables). These models were completed unadjusted and adjusted for sex, receipt of any social grant, household income, mother/caregiver education, and cognitive function as measured by standardized and time scaled Mental Processing Index and Sequential Processing score from the KABC-II [17].

DPOAE data, as an absolute DPOAE signal-to-noise ratio (SNR), in dB SPL, were used for all analyses. These data were examined at seven DPOAE frequencies spaced approximately 1000 Hz apart (1078.1, 2144.5, 3046.9, 4289.1, 5121.1, 6093.8, 7230.5 Hz). The proportion with a measurable response was calculated separately for each PHIV exposure group at each tested frequency. A linear model was applied to assess the association of PHIV exposure and DPOAE at the seven frequencies of both ears simultaneously, using generalized estimating equation (GEE) to account for the correlations of DPOAE at multiple frequencies, and allowed for different regression coefficients for different frequencies. These models were completed unadjusted and adjusted for sex, receipt of any social grant, household income, and mother/caregiver education.

ABR peak I and V latency and peak V amplitude were compared separately among the PHIV exposure groups by Kruskal–Wallis test for each ear. A linear regression model using GEE was applied to evaluate the association between PHIV exposure and ABR outcomes of both ears accounting for correlations among multiple outcomes; models were completed unadjusted and adjusted for sex, receipt of any social grant, household income, and mother/caregiver education.

The association of pure tone audiometry, DPOAE, and ABR measures with HIV disease severity in the PHIV group was assessed by corresponding linear regression models, using measures of disease severity as independent variables, adjusted for the same covariates as described above. The HIV disease severity measures included lifetime peak HIV viral load and CDC classification (converted from WHO HIV disease severity). GEE was used in all such models to account for the correlations among multiple outcomes.

## 3. Results

Three hundred and thirty-three children (105 PHIV [58 girls, 47 boys], 101 PHEU [51 girls, 50 boys], and 127 HU [65 girls, 62 boys]), mean age 11.8 years (SD = 0.6 years) had complete pure tone data; demographic characteristics of the three groups are shown in Table 1. Due to a delay in acquiring study equipment, fewer children completed DPOAE and ABR measures. A total of 284 (85%) children, 91 (87%) PHIV, 84 (83%) PHEU, and 109 (86%) HU, had complete DPOAE and ABR data. The demographic characteristics in this subset were very similar to the entire cohort (Table 1). The three groups were similar in sex and home language. Many households received some social grant across the three groups, but the proportion with household income ≤ R1000 per month ranged from 5–6% in children with PHIV to 17–20% in HU children. There were more (~20%) mothers/caregivers of children with PHIV whose highest level of education was primary school education compared to the other two groups of mothers/caregivers (~7%).

The median age [IQR] starting ART was 10.3 weeks [7.5 weeks, 28.9 weeks]. Children with PHIV had high CD4 counts (≥500 cells/mm^3^) and low HIV viral loads (<20 copies/mL) at or around the hearing testing date (Table 2), meaning that most children with PHIV were virologically controlled at the time of hearing testing. About a quarter of children did not have lifetime viral load data available and thus lifetime peak viral load was missing.

**Figure 1:**
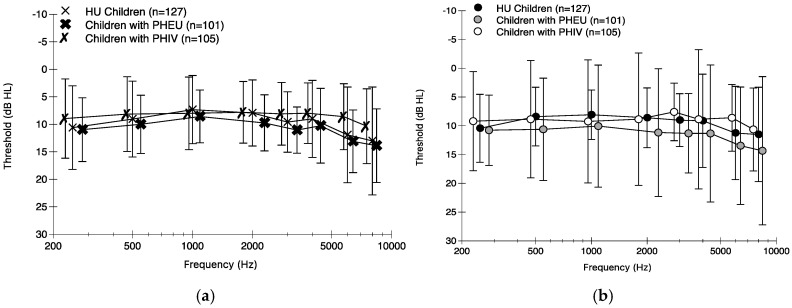
Means and standard deviations for left ear (**a**) and right ear (**b**) pure-tone thresholds for octave and interoctave frequencies are shown for HIV-unexposed (HU) children, children with perinatally HIV-exposed but unacquired (PHEU), and children with perinatally acquired HIV (PHIV).

Figure 1 displays means, and standard deviations, of pure-tone data collected for the left (top portion) and right ear (bottom portion), respectively. All three groups of children had means within normal limits (~10 dB) for both ears. Prevalence of worse ear PTA > 15 dB was slightly higher in children with PHIV (7.7%; 95% confidence interval (CI) [3.4%, 14.6%]) compared to children with PHEU (4.1%; 95% CI [1.1%, 10.1%]) and HU children (3.2%; 95% CI [0.9%, 8.0%]), but the estimates had wide confidence intervals. Children with PHIV and HU children had similar worse ear PTAs in the unadjusted model (estimate difference in mean = 0.33 dB; 95% CI [−1.98, 2.65]) and the adjusted model (estimate difference in adjusted mean = −0.02 dB; 95% CI [−2.51, 2.46]). Among children with PHIV, those with high lifetime peak viral load (>750,000 copies) had a higher worse ear PTA than those children with a lower lifetime peak viral load (≤750,000 copies) (estimate adjusted mean difference = 3.78 dB; 95% CI [−1.51, 9.07]) after adjusting for sex, receipt of social grant, household income, and caregiver education. Children with PHIV and a CDC Class C status had a higher worse ear PTA compared to children with PHIV and other class statuses (estimate adjusted mean difference = 2.91 dB; 95% CI [−1.54, 7.35]) after adjusting for sex, receipt of social grant, household income, and mother/caregiver education.

Of the 284 children with complete DPOAE data, 192 (68%) had measurable responses in the left ear and 188 (66%) had measurable responses in the right ear at all 7 frequencies. Most of the no response outcomes were from 4289 Hz and above; in fact, 93–95% of children had responses at lower frequencies (1078 and 2144 Hz), and almost 20% of children did not have measurable responses at higher frequencies (6094 and 7231 Hz). One child with PHIV had no responses at all 7 frequencies in the right ear and one HU child had no responses at all 7 frequencies in the left ear.

**Figure 2:**
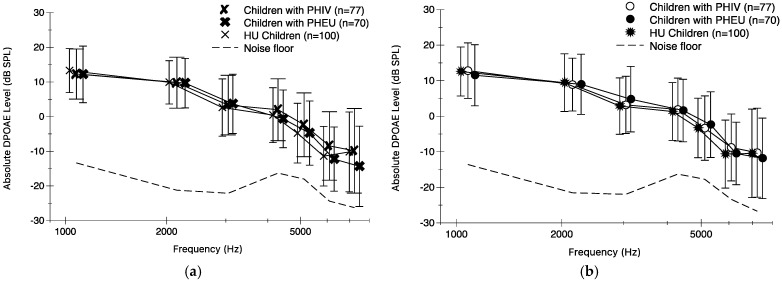
Distortion product otoacoustic emission means and standard deviations of the left ear (**a**) and right ear (**b**) are shown for children with PHIV, children with PHEU, and HU children. Mean noise floor is included as a dashed line.

Figure 2 shows absolute DPOAE means and standard deviations with the mean noise floor for the left (top portion) and right (bottom portion) ears, respectively. Mean DPOAE levels decreased with increasing frequencies for the three groups of children for both ears. There were no meaningful differences between children with PHIV compared to HU children, and children with PHEU compared to HU children, for all seven frequencies analyzed with GEE linear models after adjusting for sex, receipt of social grant, household income, and mother/caregiver education (Supplemental Table S1). Among children with PHIV, peak viral load was not associated with DPOAE levels at all frequencies except at 3046.9 Hz, where the mean DPOAE for those with a lifetime peak viral load >750,000 copies/mL was lower than those ≤750,000 copies/mL (estimate = −3.03 dB; 95% CI [−6.36, 0.30], *p* = 0.07). Also, those with CDC Class C status had similar DPOAEs across 6 of 7 frequencies compared to other CDC classes. At 1078.1 Hz, those with CDC Class C status had higher mean DPOAE compared to other CDC classes (estimate = 3.09 dB; 95% CI [0.28, 5.90], *p* = 0.03) after adjusting for sex, receipt of social grant, household income, and caregiver education.

Table 3 displays ABR data in both ears for the three groups of children. Both children with PHIV and PHEU had shorter peak I and V latencies compared to HU children, and children with PHIV had a significantly larger peak V amplitude compared to HU children. These mean differences were similar with or without adjusting for sex, receipt of social grant, household income, and mother/caregiver education (Supplemental Table S2). Lastly, among children with PHIV, no HIV disease variables (i.e., peak viral load, CDC Classification) were associated with ABR outcomes.

## 4. Discussion

There were no clinically meaningful differences in pure-tone thresholds, DPOAE, or ABR measures between children with PHIV, children with PHEU, and HU children. Children with PHIV had slightly lower median worse ear and better ear PTAs compared to children with PHEU and HU children. Children with PHIV did have a higher prevalence of worse ear PTA > 15 dB. Children with PHIV and greater HIV disease severity had slightly poorer, yet not significant, worse ear PTA outcomes compared to those with lesser HIV disease severity. Mean absolute DPOAE levels decreased with increasing frequency, but DPOAE levels were similar in the three groups. For children with PHIV, there were no significant differences in DPOAEs for HIV disease severity variables. All peak I, III, and V absolute latencies fell within normal limits, and all ABR outcomes were similar among the three groups of children. While children with PHIV and children with PHEU had statistically significant shorter peak I and V latencies than HU children, the difference estimates were not clinically meaningful. Lastly, for children with PHIV, there were no significant differences in ABR data by HIV disease severity variables. Based on data from the current study, in utero HIV acquisition or HIV exposure did not affect the outer hair cells within the cochlea and neural function up to the level of the brainstem, but children with PHIV had higher prevalence of hearing loss, and therefore it is possible there is a difference in central auditory processing.

Prevalence of worse ear hearing loss in children with PHIV in this current study (7.7%) is lower than other studies [3,4,5,12]. This may be due to this cohort starting ART earlier than earlier reports. Also, comparing the prevalence of hearing loss across studies is difficult due to the lack of a consistent definition of hearing loss. Some researchers defined hearing loss as better ear PTA > 25 dB HL [12], others have defined it as both better ear and worse ear PTA of ≥20 dB HL [3,4], and still others used a stricter definition of hearing loss as a PTA > 15 dB HL [5], which was used in the current study. Higher prevalence of hearing loss in children with PHIV may be skewed by the definition used. The prevalence of hearing loss was 24% in children with PHIV, with most (82%) having conductive hearing loss [4], which is a temporary middle ear hearing loss. The aim of the present study was to determine the prevalence of permanent hearing loss, so children were tested without any active middle ear pathology that might also affect subsequent measures, specifically DPOAEs and ABRs.

There were no differences in DPOAEs between the three groups of children, which is consistent with some earlier research [5,11] but inconsistent with other research [12]. Children with PHIV had 3–5 dB lower DPOAEs at most of the frequencies tested than HU children, but 25% of children with PHIV had abnormal tympanograms at the time of testing compared to 12% in HU children [12]. This difference in middle ear function has implications on the measurement of DPOAEs and may be the underlying cause of lower mean DPOAE levels in children with PHIV.

There were no clinically meaningful ABR measures across the three groups of children. Some have reported no difference in ABR peak V latencies for children with HIV and children without HIV [12], whereas others have reported poorer ABR outcomes in children with HIV [13,14]. In one study, however, children with HIV with an AIDS diagnosis were age-matched to children without HIV [14].

Children with PHIV and greater historical HIV disease severity had similar hearing, DPOAE, and ABR outcomes to those with lesser HIV disease severity. These findings are not consistent with previous research [3,5]. Children with PHIV and a CDC Class C disease classification had three times the odds of having hearing loss compared to children with PHIV and any other CDC disease classifications [3]. Additionally, children with PHIV and HIV RNA viral loads ≥ 400 copies/mL at time of audiometric examination were more likely to have worse DPOAEs than children with PHIV and lower HIV RNA viral loads [11]. To date, there are no studies that included the evaluation of ABR outcomes and HIV disease severity. The lack of an association between historical HIV disease severity, albeit only peak viral load and CDC Classification, and various hearing outcomes contributes to an understanding of how HIV affects auditory system development. Given that viral load is likely highest in the first year of life for children with PHIV and falls gradually over time [18], the lack of an association between peak viral load and hearing outcomes in the current study is an important finding. Children with PHIV in the current study began ART at or very close to birth, so these children were virologically and immunologically controlled based on their high mean CD4%, CD4 cell count, and mostly undetectable HIV RNA viral load at the time of audiometric testing.

A limitation of the current study is that some children with PHIV had missing data, specifically lifetime HIV viral load, so fewer children with PHIV were included in those HIV disease variable analyses. Also, most of the children with PHIV were very well virologically controlled, so there were fewer children with PHIV and greater HIV disease severity at the time of audiometric testing. Also, many children had missing congenital cytomegalovirus (CMV) data, so a determination of whether hearing loss in the current study was a consequence of congenital CMV was not possible. Congenital CMV is one of the main causes of progressive hearing loss in children [19]. Lastly, one aim of the present study was to evaluate permanent sensorineural hearing loss, so children with an active middle ear pathology were referred to medical care. Because of that, there was no opportunity to evaluate the prevalence of temporary conductive hearing loss in these three groups of children.

## 5. Conclusions

By assessing pure-tone audiometry, DPOAEs, and ABRs in children with PHIV, children with PHEU, and HU children, the results of this study contribute to the growing literature surrounding the effects of HIV in utero exposure on the auditory system. There were no clinically meaningful differences between children with PHIV, children with PHEU, and HU children in the above-mentioned audiologic measures. The lack of clinically significant differences identified in this study, however, is an important contribution to the literature such that these hearing measures may be spared the effects of HIV in utero exposure. Children with PHIV had a higher prevalence of worse ear PTA >15 dB, so audiologists should closely monitor children with PHIV for middle ear abnormalities, pure-tone thresholds, and risk factors associated with hearing loss which may impact social and educational development throughout life. Further research is needed to evaluate outcomes from reliable measures of central auditory function, such as dichotic digits testing, to assess the effects of HIV on the brainstem to cortical portions of the auditory system.

## Figures and Tables

**Table 1:** The mean and standard deviation for age and the percentage breakdown of participants’ demographics stratified by human immunodeficiency virus (HIV) infection status are shown.

CHILDREN WITH COMPLETE PURE-TONE THRESHOLDS (n = 333)			
Characteristic	PHIV (n = 105)	PHEU (n = 101)	HU (n = 127)
Age (years) mean (SD)	11.68 (0.40)	11.77 (0.56)	11.97 (0.64)
Sex at birth: Female, n (%)	58 (55%)	51 (50%)	65 (51%)
Home language Xhosa, n (%)	95 (90%)	98 (97%)	115 (91%)
Receipt of any social grant, n (%)			
Yes	91 (87%)	94 (93%)	107 (84%)
No	8 (8%)	7 (7%)	18 (14%)
Missing	6 (6%)	0 (0%)	2 (2%)
Approximate total household income, n (%) ^1^			
≤R1000 per month	6 (6%)	11 (11%)	22 (17%)
R1001–R5700 per month	85 (81%)	82 (81%)	87 (69%)
≥R5701 per month	13 (12%)	8 (8%)	17 (13%)
Mother/caregiver highest level of education completed, n (%) ^1^			
Primary school or completed primary school	22 (21%)	8 (8%)	10 (8%)
High school	56 (53%)	67 (66%)	86 (68%)
Completed high school of more than high school	26 (25%)	26 (26%)	30 (24%)
**CHILDREN WITH COMPLETE DPOAE AND ABR DATA (n = 284)**			
**Characteristic**	**PHIV (n = 91)**	**PHEU (n = 84)**	**HU (n = 109)**
Mean age, in years, (SD)	11.74 (0.40)	11.83 (0.60)	12.03 (0.67)
Sex at birth: Female, n (%)	51 (56%)	44 (52%)	57 (52%)
Home language: Xhosa, n (%)	85 (93%)	83 (99%)	108 (99%)
Receipt of any social grant, n (%)			
Yes	80 (88%)	79 (94%)	96 (88%)
No	6 (7%)	5 (6%)	11 (10%)
Missing	5 (5%)	0 (0%)	2 (2%)
Approximate total household income, n (%) ^1^			
≤R1000 per month	5 (5%)	11 (13%)	22 (20%)
R1001–R5700 per month	74 (81%)	68 (81%)	73 (67%)
≥R5701 per month	11 (12%)	5 (6%)	13 (12%)
Mother/caregiver highest level of education completed, n (%) ^1^			
Primary school or completed primary school	18 (20%)	6 (7%)	8 (7%)
High school	49 (54%)	59 (70%)	75 (69%)
Completed high school of more than high school	23 (25%)	19 (23%)	25 (23%)

^1^ One child with PHIV and one HU child had missing data for these variables.

**Table 2:** For children with PHIV, medians, interquartile ranges, and percentages are shown for HIV disease characteristics.

HIV Disease Variable	CHILDREN WITH COMPLETE PURE-TONE DATA	HIV Disease Variable	CHILDREN WITH COMPLETE DPOAE AND ABR DATA
Closest CD4 count (cells/mm^3^) (n = 97)		Closest CD4 count (cells/mm^3^) (n = 83)	
Median (Q1, Q3)	856 (645, 1029)	Median (Q1, Q3)	856 (656, 1010)
≥500	91 (87%)	≥500	79 (87%)
350–499	5 (5%)	350–499	3 (3%)
<200	1 (1%)	<200	1 (1%)
Lifetime Peak VL (copies/mL) (n = 80)		Lifetime Peak VL (copies/mL) (n = 66)	
<750,000	25 (24%)	<750,000	22 (24%)
>750,000	55 (52%)	>750,000	44 (48%)
Closest VL (copies/mL) (n = 104)		Current VL (copies/mL) (n = 90)	
≤20 ^a^	78 (74%)	≤20 ^1^	67 (74%)
<40	5 (5%)	<40	5 (5%)
<100	14 (13%)	<100	11 (12%)
<400	4 (4%)	<400	4 (4%)
≥400	3 (3%)	≥400	3 (3%)
CDC class C, (n = 103), Yes, n (%)	43 (41%)	CDC class C, (n = 89), Yes, n (%)	37 (41%)

VL: viral load; mm^3^: millimeters cubed; mL: milliliter. ^1^ Categories are exclusive and labeled by the different detectable levels from different data reports.

**Table 3:** Means and standard deviations for right and left ear auditory brainstem response (ABR) peak I, III, and V absolute latencies; I–III and I–V interpeak latencies; and peak V amplitude for children with PHIV, children with PHEU, and HU children.

RIGHT EAR						
	Peak I Latency (msec) Mean (SD)	Peak III Latency (msec) Mean (SD)	Peak V Latency (msec) Mean (SD)	I–III Interpeak Latency (msec) Mean (SD)	I–V Interpeak Latency (msec) Mean (SD)	Peak V Amplitude ^1^ (μV) Mean (SD)
PHIV (N = 85)	1.55 (0.12)	3.69 (0.15)	5.53 (0.22)	2.15 (0.14)	3.99 (0.23)	0.46 (0.20)
PHEU (N = 77)	1.60 (0.13)	3.74 (0.18)	5.61 (0.23)	2.15 (0.18)	4.01 (0.22)	0.41 (0.15)
HU (N = 104)	1.64 (0.16)	3.78 (0.18)	5.65 (0.21)	2.14 (0.17)	4.01 (0.19)	0.38 (0.15)
**LEFT EAR**						
PHIV (N = 85)	1.54 (0.13)	3.70 (0.19)	5.49 (0.24)	2.16 (0.15)	3.96 (0.22)	0.48 (0.18)
PHEU (N = 79)	1.56 (0.12)	3.72 (0.15)	5.53 (0.22)	2.15 (0.15)	3.96 (0.23)	0.42 (0.16)
HU (N = 104)	1.61 (0.14)	3.75 (0.20)	5.63 (0.28)	2.14 (0.20)	4.01 (0.23)	0.40 (0.15)

PHIV: perinatally acquired human immunodeficiency virus; PHEU: perinatally human immunodeficiency virus-exposed but uninfected; HU: human immunodeficiency virus-unexposed; msec: millisecond; μV: microvolt; SD: standard deviation. ^1^ Peak V Amplitude sample size was as follows: PHIV (n = 75); PHEU (n = 76); and HU (n = 103) for both ears.

## Data Availability

The original data presented in this study are available on reasonable request from the corresponding author. The data are not publicly available due to privacy concerns.

## Supplementary Materials

The following supporting information can be downloaded at https://www.mdpi.com/article/10.3390/audiolres15060170/s1, Table S1: GEE linear models for the association of HIV status and each DPOAE frequency; Table S2: GEE linear models for the association of HIV status and ABR outcomes.

## Abbreviations

The following abbreviations are used in this manuscript:

HIV	human immunodeficiency virus
PHIV	perinatally acquired HIV
PHEU	perinatal HIV exposure, but uninfected
HU	HIV-unexposed
DPOAE	distortion product otoacoustic emissions
ABR	auditory brainstem response
ART	antiretroviral therapy
PTA	pure-tone average
GEE	generalized estimating equation
