# Supplementary material for: Hearing Abilities in Children with Perinatally Acquired HIV, Children Perinatally Exposed to HIV but Uninfected, and Children Unexposed to HIV
Source: Audiol Res. 2025 Dec 5;15(6):170. doi: 10.3390/audiolres15060170 (PMC12729991; doi:10.3390/audiolres15060170)
Supplement: Supplementary file 1 [file audiolres-15-00170-s001.zip › audiolres-3902439-supplementary.pdf]

Supplemental Table S1. GEE linear models for the association of HIV status and each DPOAE frequency.

DPOAE Frequency (Hz)	HIV Group	UNADJUSTED Difference Estimate	UNADJUSTED 95% Confidence Interval	UNADJUSTED p-value		ADJUSTED* Difference Estimate	ADJUSTED* 95% Confidence Interval	ADJUSTED* p-value
1078.1	PHIV vs HU	-0.39	-2.31 to 1.53	0.69		-0.49	-2.55 to 1.56	0.64
	PHEU vs HU	-0.59	-2.51 to 1.34	0.55		-0.46	-2.41 to 1.48	0.64
2144.5	PHIV vs HU	-0.45	-2.56 to 1.66	0.68		-0.48	-2.74 to 1.77	0.67
	PHEU vs HU	-0.12	-2.27 to 2.04	0.91		0.11	-2.08 to 2.30	0.92
3046.9	PHIV vs HU	1.06	-1.20 to 3.32	0.36		1.19	-1.15 to 3.54	0.32
	PHEU vs HU	1.65	-0.82 to 4.12	0.19		1.95	-0.53 to 4.43	0.12
4289.1	PHIV vs HU	1.18	-0.96 to 3.31	0.28		1.28	-0.92 to 3.48	0.25
	PHEU vs HU	-1.01	-3.24 to 1.22	0.37		-0.68	-2.90 to 1.55	0.55
5121.1	PHIV vs HU	0.47	-1.93 to 2.87	0.70		0.72	-1.76 to 3.20	0.57
	PHEU vs HU	0.11	-2.23 to 2.46	0.93		0.46	-1.87 to 2.79	0.70
6093.8	PHIV vs HU	1.73	-0.66 to 4.11	0.16		1.87	-0.61 to 4.35	0.14
	PHEU vs HU	-0.89	-3.27 to 1.50	0.47		-0.56	-2.97 to 1.85	0.65
7230.5	PHIV vs HU	0.94	-2.04 to 3.91	0.54		1.14	-1.89 to 4.16	0.46
	PHEU vs HU	-2.37	-5.28 to 0.54	0.11		-1.99	-4.90 to 0.93	0.18

*Adjusted for sex, receipt of social grant, household income, and caregiver education
DPOAE: distortion product otoacoustic emission; PHIV: perinatally-acquired human immunodeficiency virus; PHEU: perinatally human immunodeficiency virus-exposed but uninfected; HU: human immunodeficiency virus-unexposed.

Supplemental Table S2. GEE linear models for the association of HIV status and ABR outcomes.

		UNADJUSTED				ADJUSTED*		
**ABR Outcome**	**HIV Group**	**Difference Estimate**	**95% Confidence Interval**	**p-value**		**Difference Estimate**	**95% Confidence Interval**	**p-value**
Peak I latency (msec)	PHIV vs HU	-0.08	-0.12 to -0.05	<0.001		-0.08	-0.12 to -0.05	<0.001
	PHEU vs HU	-0.04	-0.08 to -0.01	0.03		-0.04	-0.08 to -0.01	0.02
Peak V latency (msec)	PHIV vs HU	-0.12	-0.18 to -0.06	<0.001		-0.13	-0.19 to -0.07	<0.001
	PHEU vs HU	-0.07	-0.13 to -0.01	0.03		-0.07	-0.13 to -0.01	0.03
Peak V amplitude (μV)	PHIV vs HU	0.08	0.03 to 0.13	0.001		0.08	0.03 to 0.13	0.001
	PHEU vs HU	0.02	-0.02 to 0.06	0.28		0.02	-0.02 to 0.06	0.32

*Adjusted for sex, receipt of social grant, household income, and caregiver education

ABR: auditory brainstem response; PHIV: perinatally-acquired human immunodeficiency virus; PHEU: perinatally human immunodeficiency virus-exposed but uninfected; HU: human immunodeficiency virus-unexposed.
